# A novel tetrahedral framework nucleic acid‐derived chemodynamic therapy agent for effective glioblastoma treatment

**DOI:** 10.1111/cpr.13736

**Published:** 2024-08-24

**Authors:** Xiaodie Li, Lei Li, Xin Fu, Shiqian Huang, Yuhao Wang, Yuepeng Yang, Shuqin Zhou, Zhaowei Zou, Qing Peng, Chao Zhang

**Affiliations:** ^1^ Department of Oncology, Zhujiang Hospital Southern Medical University Guangzhou China; ^2^ Department of General Surgery, Zhujiang Hospital Southern Medical University Guangzhou China; ^3^ Clinical Research Center, Zhujiang Hospital Southern Medical University Guangzhou China; ^4^ Department of Anesthesiology of The Second Affiliated Hospital, School of Medicine The Chinese University of Hong Kong, Shenzhen & Longgang District People's Hospital of Shenzhen Shenzhen China; ^5^ Central Laboratory of The Second Affiliated Hospital, School of Medicine The Chinese University of Hong Kong, Shenzhen & Longgang District People's Hospital of Shenzhen Shenzhen China

## Abstract

Chemodynamic therapy (CDT) has garnered significant attention for treating diverse malignant tumours due to its minimally invasive nature, reduced damage to healthy tissues, and potential mitigation of side effects. However, its application in glioblastoma (GBM) is hindered by the diminished capacity of CDT agents to traverse the blood–brain barrier (BBB), inadequate tumour targeting efficiency, and restricted availability of H_2_O_2_ within the tumour microenvironment (TME). To address these challenges, we devised a novel CDT agent (Fe@tFNAs‐ANG‐3AT) based on a tetrahedral framework nucleic acids (tFNAs). Fe@tFNAs‐ANG‐3AT was constructed by anchoring iron ions (Fe^3+^) onto the dual appendages‐modified tFNAs. Specifically, one appendage, Angiopep‐2 (ANG, a penetrating peptide), facilitates Fe@tFNAs‐ANG‐3AT penetration across the BBB and selective targeting of tumour cells. Simultaneously, the second appendage, 3‐Amino‐1,2,4‐triazole (3AT, a H_2_O_2_ enzyme inhibitor), augments the H_2_O_2_ levels required for effective CDT treatment. Upon tumour cell internalization, the loaded Fe^3+^ in Fe@tFNAs‐ANG‐3AT is reduced to Fe^2+^ by the overexpressed glutathione (GSH) in the TME, catalysing the generation of cytotoxic hydroxyl radicals (·OH) and inducing tumour cell death via elevated oxidative stress levels within tumour cells. It is anticipated that Fe@tFNAs‐ANG‐3AT holds promise as a transformative treatment strategy for GBM.

## Graphical abstract

A novel CDT agent (Fe@tFNAs‐ANG‐3AT) is developed and demonstrates the capability to effectively cross the BBB and selectively accumulate in tumour cells. Upon tumour cell internalization, Fe@tFNAs‐ANG‐3AT can augment the H_2_O_2_ levels and react with GSH and H_2_O_2_ to generate •OH, thus leading to an effective CDT against GBM.

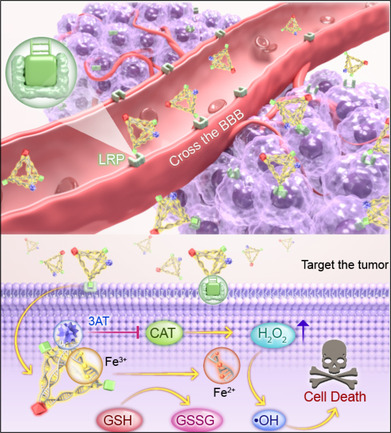

## 1 INTRODUCTION

Glioblastoma (GBM) is the most aggressive and fatal central nervous system tumour.^1^ Surgical removal of tumour tissue, chemotherapy, and radiotherapy are common clinical treatment methods for GBM.^2^, ^3^, ^4^ However, the high infiltrative and invasive nature of GBM cells makes precise tumour resection difficult.^5^ Additionally, due to the hypoxic microenvironment and deep intracerebral location of GBM tumours as well as the presence of the highly restrictive blood–brain barrier (BBB) and poor specificity in targeting tumour cells,^6^, ^7^, ^8^, ^9^ the clinical therapeutic effect of radiotherapy and chemotherapy is also markedly hampered. Given these limitations of GBM treatment, the treatment strategy that chemotherapeutic agents combined with targeted therapies has been widely developed in recent years, but still faces the challenges of drug resistance, tumour heterogeneity, cost and potential side effects.^10^ Therefore, it is urgent to explore novel therapeutic approaches to ameliorate the current situation for GBM treatment.

In recent years, chemodynamic therapy (CDT) has gained significant attention in the treatment of various malignant tumours, primarily attributed to the following unique advantages: First, CDT is minimally invasive, minimizing damage to normal tissues and potential side effects. Additionally, CDT uses transition metal ions, such as manganese (Mn^2+^), ferrous (Fe^2+^), and copper (Cu^2+^) ions, as catalysts to convert endogenous H_2_O_2_ into highly reactive hydroxyl radicals (·OH) through Fenton or Fenton‐like reactions, thereby increasing oxidative stress levels within tumour cells to induce tumour cells death.^11^, ^12^, ^13^, ^14^, ^15^ Moreover, in contrast to other dynamic therapies (encompassing sono‐dynamic, photodynamic, and electrodynamic), CDT is not affected by external stimuli (such as sound, light, and electricity) and is independent of a continuous oxygen supply,^16^, ^17^ showing significant spatiotemporal selectivity. These notable attributes of CDT empower it to surmount the obstacles posed by penetration depth and hypoxic microenvironment, rendering it a promising avenue for treating GBM.^18^, ^19^, ^20^, ^21^ Nevertheless, the highly restrictive BBB impedes the entry of CDT agents (such as manganese, iron, and copper) into brain tissues and tumour sites, resulting in inadequate accumulation at the tumour site. In addition, the insufficient endogenous H_2_O_2_ concentration in the tumour microenvironment (TME) also limits the sustained killing ability of CDT on tumour cells. These limitations hinder the efficacy of CDT for GBM. Therefore, it is crucial to explore suitable strategies and carriers to promote the penetration of CDT agents across the BBB, enhance their targeting ability towards tumour cells, and increase the H_2_O_2_ content in the TME to improve the efficacy of CDT for GBM.

In recent years, due to the wide range of biological functions of DNA nanostructures, DNA frameworks‐based nucleic acid nanostructures have been designed and synthesized for the treatment of various diseases.^22^, ^23^, ^24^, ^25^, ^26^, ^27^ Among these, tetrahedral framework nucleic acids (tFNAs), as a novel three‐dimensional nucleic acid nanomaterial, have been widely developed and used to construct novel pharmaceuticals that perform specific functions based on it in the field of biomedicine.^28^ On the one hand, tFNAs have good biocompatibility, low immunogenicity, and structural stability.^29^ On the other hand, tFNAs have unparalleled programmability and can be precisely assembled with diverse drugs or modified with functional units such as targeting peptides, antibodies, and nucleic acid aptamers.^30^, ^31^ Therefore, many tFNAs complex with specific functions can be formed by processing and modifying tFNAs. This characteristic is expected to enhance their ability to penetrate the BBB and specifically target tumour cells, as well as increase the H_2_O_2_ content required for exerting the efficacy of CDT agents in the TME. Consequently, using tFNAs as nanocarriers for CDT agents shows great potential in combating GBM. However, to the best of our knowledge, this research field is still relatively blank.

**FIGURE 1:**
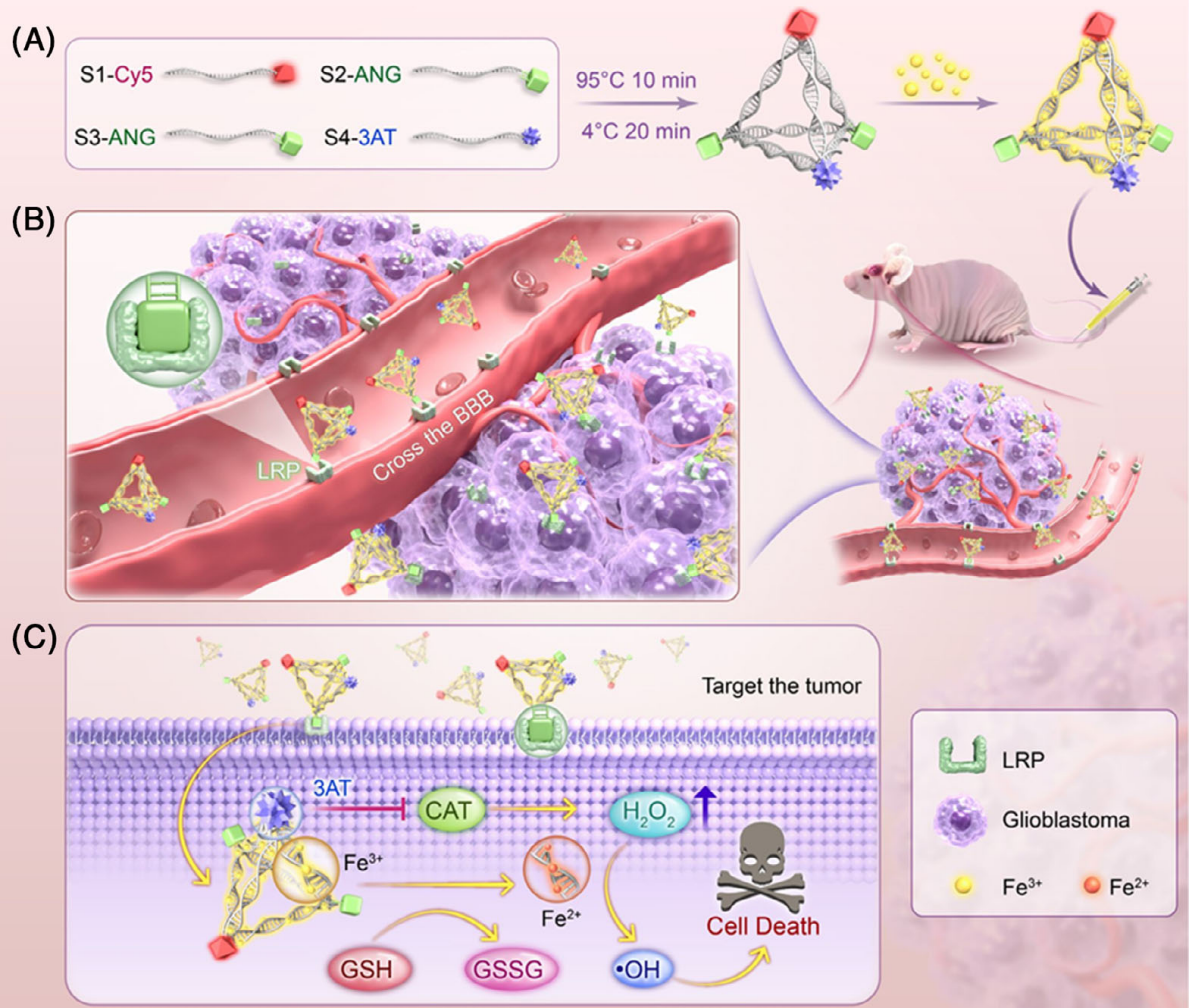
Scheme of the thorough synthesis pathway and functional mechanism of the Fe@tFNAs‐ANG‐3AT. (A) The design and preparation of Fe@tFNAs‐ANG‐3AT. (B) Schematic illustration of Fe@tFNAs‐ANG‐3AT crossing the BBB to accumulate in GBM after intravenous administration. (C) The mechanism of Fe@tFNAs‐ANG‐3AT achieving active tumour uptake and catalysing CDT.

In this study, to promote the efficiency of CDT treatment against GBM, we designed a novel CDT agent (Fe@tFNAs‐ANG‐3AT) by loading Fe^3+^ onto double‐appendage modified tFNAs (Figure 1A). This CDT agent not only has great ability to cross the BBB and target tumour cells but also enhances the H_2_O_2_ content necessary for exerting the efficacy of CDT agents in the TME, thus significantly improving the anti‐tumour efficacy of CDT against GBM, which is distinct from other CDT agents in previous studies. Specifically, on the one hand, one of the appendages, Angiopep‐2 (ANG, a penetrating peptide) binds specifically to the highly expressed low‐density lipoprotein receptor‐related protein (LRP) on the surface of brain capillary endothelial cells and tumour cells,^32^, ^33^ promoting the penetration of Fe@tFNAs‐ANG‐3AT through the BBB and precise targeting of tumour cells (Figure 1B). Imaging results showed that Fe@tFNAs‐ANG‐3AT successfully crossed the BBB and accumulated in tumour tissue. On the other hand, the second appendage, 3‐Amino‐1,2,4‐triazole (3AT, a H_2_O_2_ enzyme inhibitor) inhibits the decomposition of H_2_O_2_ in the TME, increasing the H_2_O_2_ levels required for Fe@tFNAs‐ANG‐3AT to exert CDT efficacy,^34^ achieving sustained and stable killing ability of Fe@tFNAs‐ANG‐3AT on tumour cells. Subsequently, the loaded Fe^3+^ in Fe@tFNAs‐ANG‐3AT was reduced to Fe^2+^ by glutathione (GSH) overexpressed in the TME, catalysing the formation of cell‐toxic ·OH, inducing tumour cells death by increasing the oxidative stress levels of the tumour cells (Figure 1C). Moreover, safety evaluation results show that Fe@tFNAs‐ANG‐3AT possesses excellent biosafety. In conclusion, our study proposes a safe and promising approach for treating GBM, which may provide a prospective therapeutic strategy for clinical treatment of GBM and is expected to improve patient outcomes in the future.

## 2 RESULTS AND DISCUSSION

### 2.1 Preparation and characterization of Fe@tFNAs‐ANG‐3AT

**FIGURE 2:**
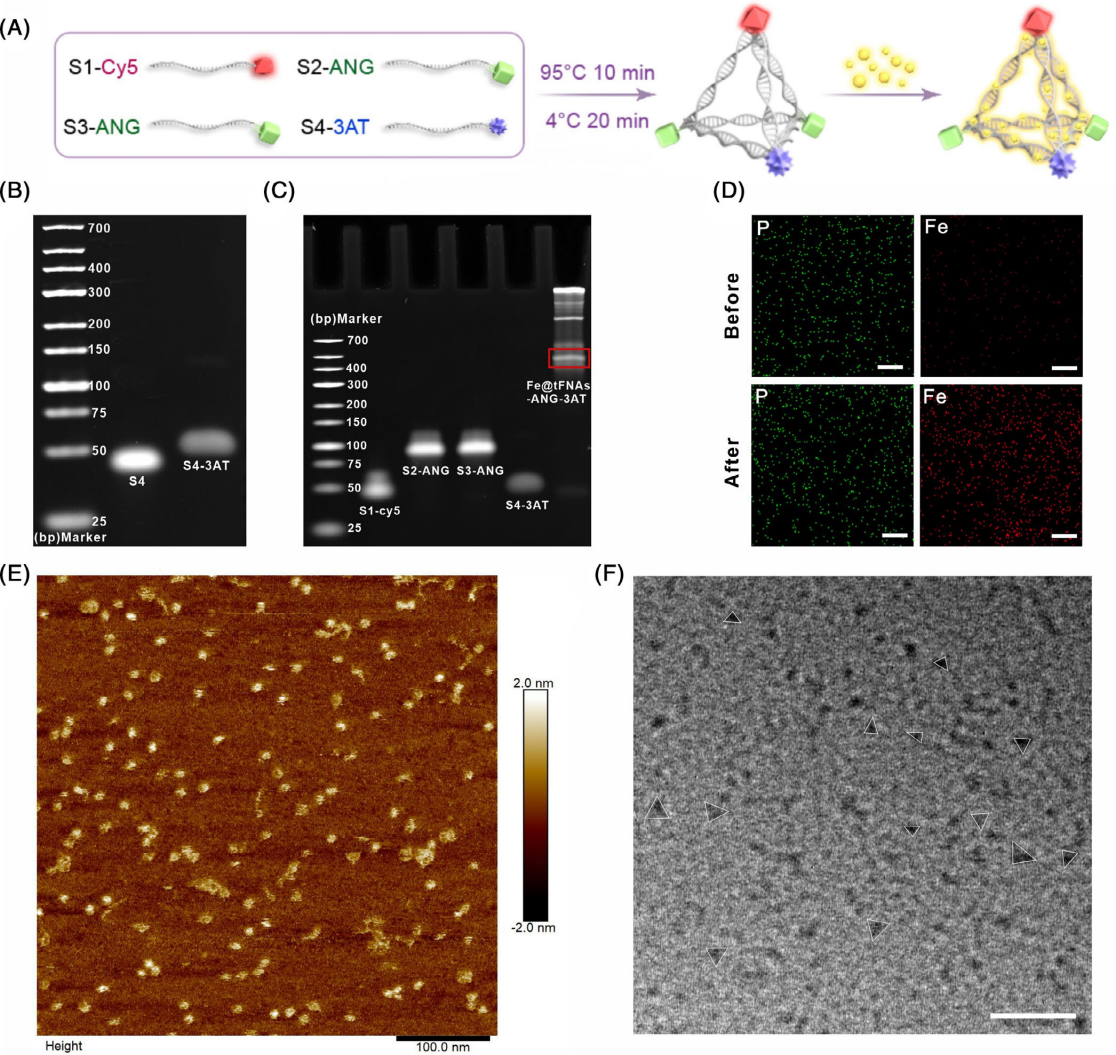
Synthesis and Characterization of Fe@tFNAs‐ANG‐3AT. (A) Schematic diagram of Fe@tFNAs‐ANG‐3AT fabrication. (B, C) 8% PAGE analysis of the successful synthesis of S4‐3AT and Fe@tFNAs‐ANG‐3AT. (D) EDS element maps for P and Fe of tFNAs‐ANG‐3AT and Fe@tFNAs‐ANG‐3AT. Scale bars: 10 μm. (E) AFM image of Fe@tFNAs‐ANG‐3AT. Scale bar: 100 μm. (F) TEM image of Fe@tFNAs‐ANG‐3AT. Scale bar: 50 μm.

In our study, we performed structural modification of tFNAs to create a drug delivery system capable of promoting the accumulation of CDT drugs in GBM tumour tissue and exerting sustained and stable anti‐tumour effects. Specifically, 3AT, an H_2_O_2_ enzyme inhibitor, was grafted onto the 5′ end of S4 ssDNA (S4‐3AT) (Figure 2A). 8% polyacrylamide gel electrophoresis (PAGE) showed that compared with S4 ssDNA, S4‐3AT had a larger molecular weight and migrated more slowly during gel electrophoresis (Figure 2B), indicating successful grafting of 3AT onto S4 ssDNA. Additionally, the fluorescent molecule Cy5 was grafted onto the 5′ end of S1 ssDNA (S1‐Cy5) for subsequent analysis of the localization of Fe@tFNAs‐ANG‐3AT. ANG peptide, a penetrating peptide, was separately grafted onto the 5′ end of S2 ssDNA and S3 ssDNA (S2‐ANG and S3‐ANG). Subsequently, these grafted modifications of ssDNA self‐assembled into tFNAs‐ANG‐3AT using a one‐pot annealing method (Figure 2A). 8% PAGE showed that among these DNA single strands and complex, Fe@tFNAs‐ANG‐3AT had the largest molecular weight and migrated at the slowest speed (Figure 2C), confirming the successful synthesis of Fe@tFNAs‐ANG‐3AT, which was further testified by dynamic light scattering (DLS) (Figure S1). Next, as shown in Figure 2D, elemental mapping data showed that compared with tFNAs‐ANG‐3AT, Fe@tFNAs‐ANG‐3AT had a significantly increased content of iron (Fe) element, whereas there was no significant difference in phosphorus (P) element content, demonstrating successful loading of Fe^3+^ onto tFNAs‐ANG‐3AT. Finally, the microstructure of Fe@tFNAs‐ANG‐3AT was characterized using atomic force microscopy (AFM) and transmission electron microscopy (TEM), and the results showed that the synthesized Fe@tFNAs‐ANG‐3AT had a triangular structure (Figure 2E,F), further confirming the successful self‐assembly of Fe@tFNAs‐ANG‐3AT.

### 2.2 Evaluation of the BBB crossing ability and tumour targeting ability of Fe@tFNAs‐ANG‐3AT

**FIGURE 3:**
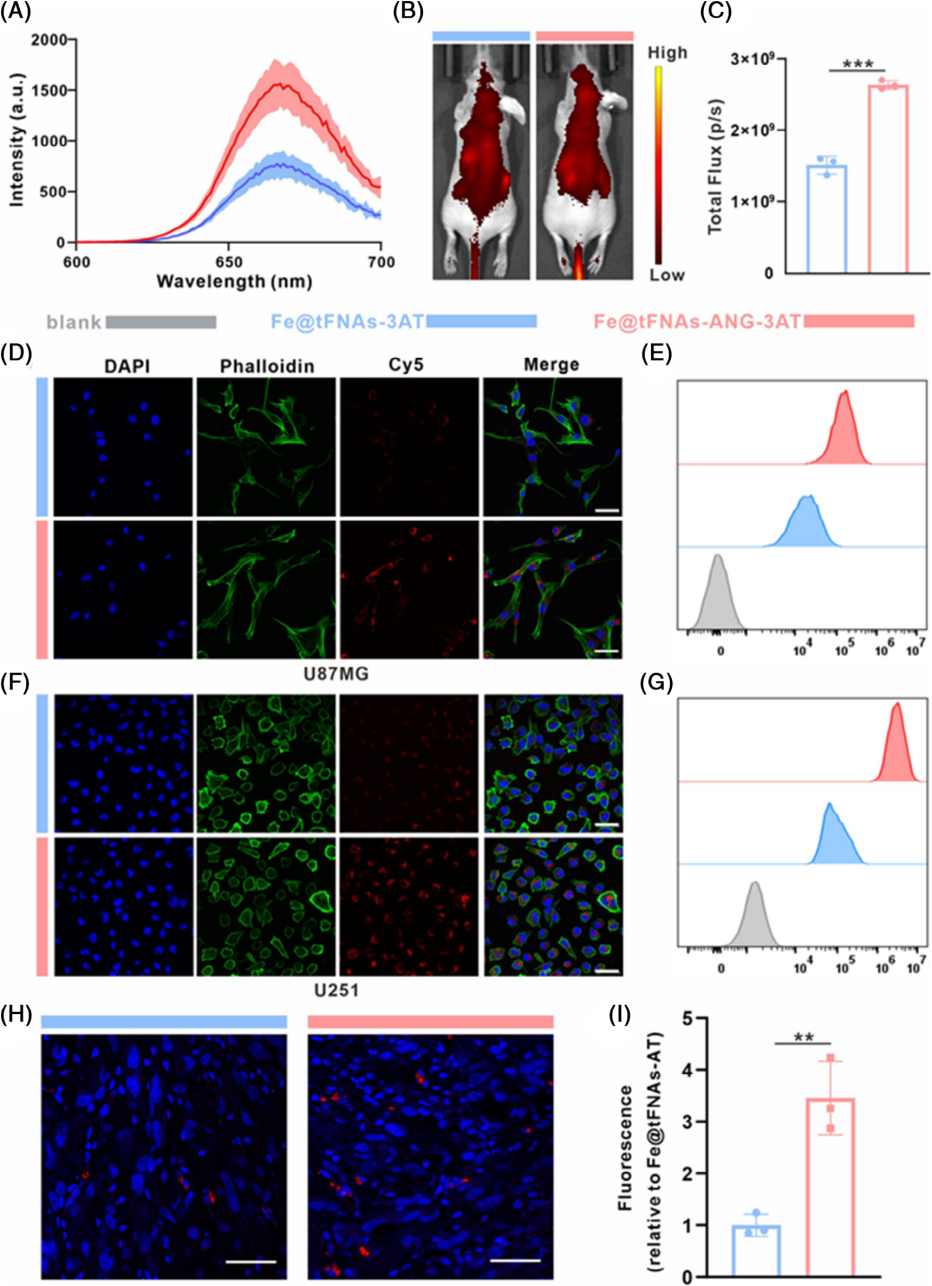
Investigation of the BBB‐traversing and GBM‐targeting ability of Fe@tFNAs‐ANG‐3AT. (A) The fluorescence spectroscopy analysis of Cy5‐labelled Fe@tFNAs‐3AT and Fe@tFNAs‐ANG‐3AT in in vitro BBB model. (B) In vivo fluorescent images after intravenous administration with Cy5‐labelled Fe@tFNAs‐3AT and Fe@tFNAs‐ANG‐3AT for 30 min in normal mice. (C) Quantitative analysis of the fluorescent intensity of brains. Data are presented as the mean ± SD (*n* = 3, ***p < 0.001). (D, E) CLSM images (D) and flow cytometry analysis (E) of U87MG cells treated with indicated samples. (F, G) CLSM images (F) and flow cytometry analysis (G) of U251 cells treated with indicated samples. (H) immunofluorescence images of the tumour slice in the indicated groups. Scale bars: 50 μm. (I) Quantitative assessment of the relative fluorescent intensity of tumour slice. Data are presented as the mean ± SD (*n* = 3, **p < 0.01).

To investigate the BBB permeability of Fe@tFNAs‐ANG‐3AT, we designed a transwell model based on mouse brain microvascular endothelial cells (bEnd.3). Fluorescence quantification analysis showed that compared with the Cy5‐Fe@tFNAs‐3AT group (Cy5‐labelled Fe@tFNAs‐3AT), the Cy5‐Fe@tFNAs‐ANG‐3AT group (Cy5‐labelled Fe@tFNAs‐ANG‐3AT) exhibited significantly increased fluorescence intensity in the bottom culture medium (~2‐fold) (Figures 3A and S2), indicating that Fe@tFNAs‐ANG‐3AT effectively penetrated the dense endothelial cell layer mimicking the BBB due to the specific recognition of LRP highly expressed on the surface of brain capillary endothelial cells by ANG peptide. In addition, we also investigated the in vivo BBB permeability using normal nude mice. Cy5‐Fe@tFNAs‐3AT and Cy5‐Fe@tFNAs‐ANG‐3AT were administered to mice intravenously through the tail vein, and the fluorescence intensity of Cy5 in the mice brain was monitored in real time using an in vivo imaging system. The fluorescence images showed stronger fluorescence in the Cy5‐Fe@tFNAs‐ANG‐3AT group in the mice brain, and the fluorescence intensity was roughly 1.7 times greater than that observed in the Cy5‐Fe@tFNAs‐3AT group (Figure 3B,C). These results suggest that the ANG peptide in Fe@tFNAs‐ANG‐3AT can specifically bind to LRP highly expressed on the surface of brain capillary endothelial cells, significantly improving the BBB permeability efficiency of the nanosystem.

To evaluate the targeting ability of Fe@tFNAs‐ANG‐3AT towards tumour cells, we stained the cell cytoskeleton and nucleus using Phalloidin and DAPI respectively, and detected the uptake of the nanoparticle system by U251 and U87 GBM cells after 6 h of incubation using confocal microscopy and flow cytometry. Confocal imaging and fluorescence co‐localization analysis showed that compared with the Cy5‐Fe@tFNAs‐3AT group, the red fluorescence of Cy5‐Fe@tFNAs‐ANG‐3AT group in the cytoplasm of U251 and U87MG cells was significantly enhanced, indicating significantly increased uptake of Cy5‐Fe@tFNAs‐ANG‐3AT by both GBM cell lines (Figures 3D,F, S3, and S4). Similar results were observed in flow cytometry analysis as shown in Figure 3E,G. Subsequently, we further investigated the tumour targeting ability of Fe@tFNAs‐ANG‐3AT in a GBM mouse model orthotopically. Ex vivo imaging results indicated superior tumour‐targeting capability of Fe@tFNAs‐ANG‐3AT over Fe@tFNAs‐3AT (Figure S5). Immunofluorescence staining of excised tumour tissues showed that the red fluorescence intensity in the tumour tissues of Cy5‐Fe@tFNAs‐ANG‐3AT group was significantly higher than that of Cy5‐Fe@tFNAs‐3AT group after intravenous administration (Figure 3H,I), indicating stronger tumour targeting ability and enhanced tumour accumulation of Cy5‐Fe@tFNAs‐ANG‐3AT. These experimental results fully confirmed that the ANG peptide in Fe@tFNAs‐ANG‐3AT can specifically bind to LRP overexpressed on the surface of tumour cells, thereby exhibiting excellent targeting ability towards GBM cells and tumour tissues and helping to improve the accumulation concentration of nanoparticles in tumour tissues.

### 2.3 In vitro anticancer effect of Fe@tFNAs‐ANG‐3AT against GBM

**FIGURE 4:**
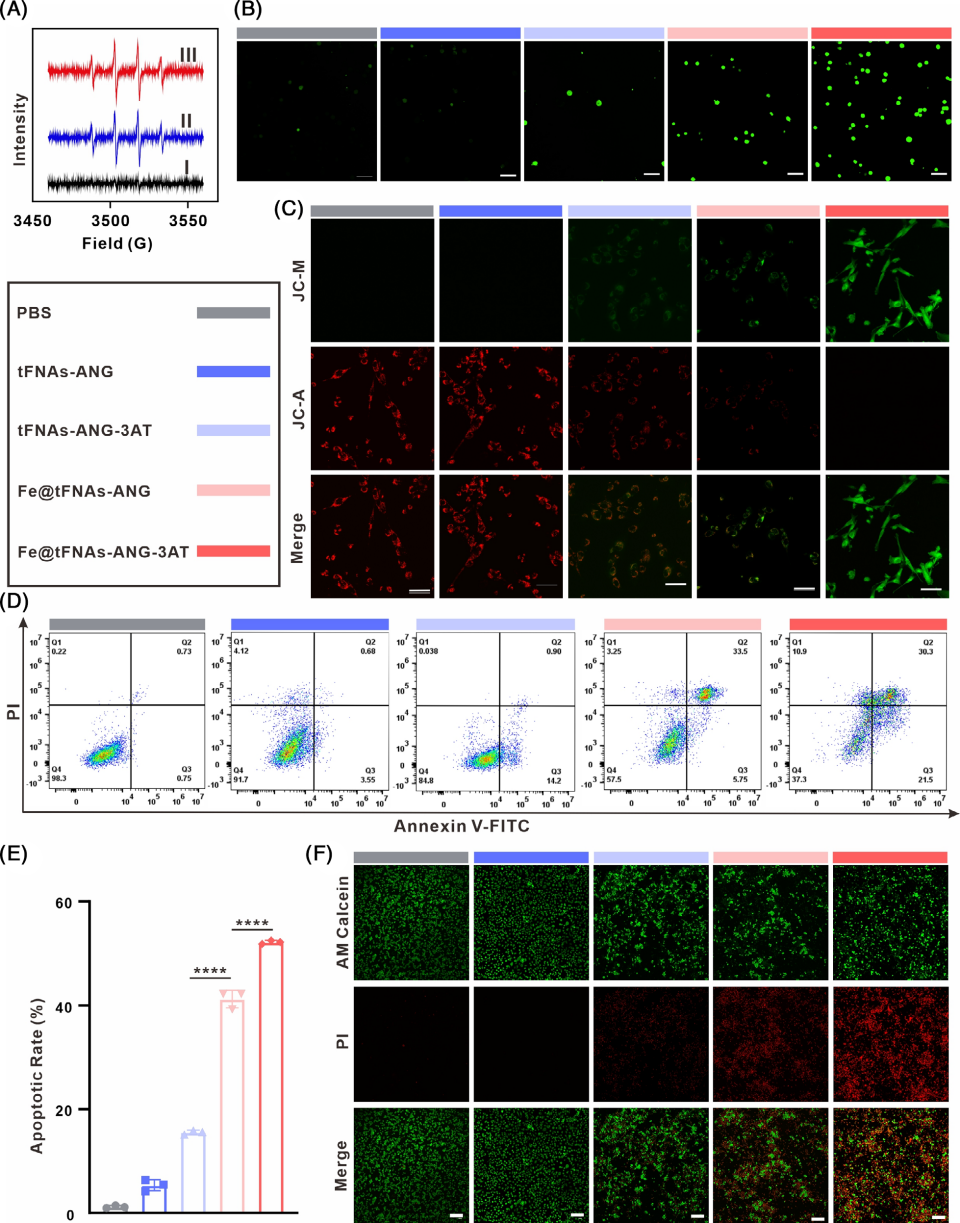
In vitro anticancer effect of Fe@tFNAs‐ANG‐3AT‐rendered CDT against GBM. (A) ESR spectra of different reaction systems with DMPO as the spin trap. (I: Catalase (+), 3AT (−); II: Catalase (−), 3AT (−); III: Catalase (+), 3AT (+)). (B, C) CLSM images analysis of ROS generation (B) and JC‐1 staining (C) in U87MG cells after indicated treatments. Scale bars: 50 μm. (D, E) Cell apoptosis analysed by flow cytometry (D) and apoptotic rate in U87MG cells after indicated administration. Results are depicted as the mean ± SD (*n* = 3, ****p < 0.0001). (F) CLSM images analysis of Live&dead staining, scale bars: 200 μm.

To investigate the ability of Fe@tFNAs‐ANG‐3AT‐mediated CDT treatment to generate cytotoxic ·OH (a type of ROS), we first evaluated the ability of the CDT agent to generate ·OH at the solution level by electron spin resonance (ESR) spectroscopy with 5,5‐dimethyl‐1‐pyrroline N‐oxide (DMPO) as a ·OH trapping agent. As shown in Figure 4A, due to the presence of GSH and H_2_O_2_ in group ΙΙ, Fe^3+^ in Fe@tFNAs‐ANG‐3AT is reduced to Fe^2+^ by GSH, and then Fe^2+^ catalysed the production of a large amount of ·OH through the Fenton reaction, resulting in a strong ·OH signal being detected. When catalase was added in group I, the Fenton reaction was suppressed due to the absence of H_2_O_2_, resulting in only a weak ·OH signal. This situation was turned around in the presence of the catalase inhibitor 3AT (group III), as 3AT restored the levels of H_2_O_2_ in the solution due to its catalase inhibition activity, leading to a strong ·OH signal again. TMB was also used as an indicator of ·OH to evaluate the ability of the CDT agent to produce ·OH, which obtained the results consistent with the aforementioned (Figure S6). Subsequently, we evaluated the levels of ·OH produced by the CDT agent in U87MG cells using the ROS fluorescent probe 2,7‐dichlorofluorescin diacetate (DCFH‐DA). As shown in Figure 4B, PBS, tFNAs‐ANG, and tFNAs‐ANG‐3AT had no effect on ·OH production. Due to the Fe^2+^ catalysed Fenton reaction, a small amount of ·OH was generated in GBM cells treated with Fe@tFNAs‐ANG. In contrast, the GBM cells treated with Fe@tFNAs‐ANG‐3AT exhibited a notable elevation in green fluorescence, indicating heightened levels of ·OH production. This is attributed to the ability of 3AT in Fe@tFNAs‐ANG‐3AT to inhibit the decomposition of H_2_O_2_ in the TME, thereby providing sufficient H_2_O_2_ for the Fe^2+^ catalysed Fenton reaction and significantly increasing the intracellular ·OH levels. Notably, mitochondria are key organelles that are highly sensitive to reactive oxygen species (ROS).^35^, ^36^ 5,5′,6,6′‐Tetrachloro‐1,1′,3,3′‐tetraethyl‐imidacarbocyanine iodide (JC‐1), a fluorescent probe, was used to evaluate alterations in mitochondrial membrane potential in U87MG cells. As shown in Figure 4C, the GBM cells treated with Fe@tFNAs‐ANG‐3AT exhibited a stronger green fluorescence (JC‐1 monomers, JC‐M) compared with the other groups, whereas the red fluorescence (JC‐1 aggregates, JC‐A) was almost absent, indicating a significant decrease in intracellular mitochondrial membrane potential due to the large amount of ·OH production. These results confirm that Fe@tFNAs‐ANG‐3AT is able to increase the required levels of H_2_O_2_ for its CDT treatment, which in turn generates a large amount of ·OH through the Fenton reaction and affects mitochondrial function by inducing mitochondrial membrane oxidation.

Next, we evaluated the ability of CDT treatment mediated by Fe@tFNAs‐ANG‐3AT to induce apoptosis in U87MG cells using Annexin V‐FITC and PI staining. As shown in Figure 4D,E, the level of cell apoptosis in GBM cells treated with tFNAs‐ANG and tFNAs‐ANG‐3AT was negligible, indicating that the blank vectors tFNAs‐ANG and tFNAs‐ANG‐3AT demonstrated excellent biocompatibility and had no oxidative damage to U87MG cells. Furthermore, compared with Fe@tFNAs‐ANG, Fe@tFNAs‐ANG‐3AT exhibited significantly enhanced killing ability in U87MG cells, with a cell apoptosis rate of approximately 52%. This can be attributed to the presence of 3AT in Fe@tFNAs‐ANG‐3AT, which can increase the H_2_O_2_ levels required for CDT treatment, thereby generating more cytotoxic ·OH and achieving sustained and stable killing of GBM cells. The results of live/dead cell staining (Figure 4F) demonstrated that Fe@tFNAs‐ANG‐3AT possessed efficient ·OH generation ability, with lower cell viability compared with the other groups, which aligned with the results of the cell apoptosis assay. Taken together, these experimental results demonstrate that Fe@tFNAs‐ANG‐3AT can efficiently generate cytotoxic ·OH inside tumour cells, thereby increasing intracellular oxidative stress levels and inducing cell death, indicating that Fe@tFNAs‐ANG‐3AT, as a promising CDT agent, possesses excellent antitumor effects in vitro.

### 2.4 Anti‐tumour efficacy of Fe@tFNAs‐ANG‐3AT on orthotopic GBM mice

**FIGURE 5:**
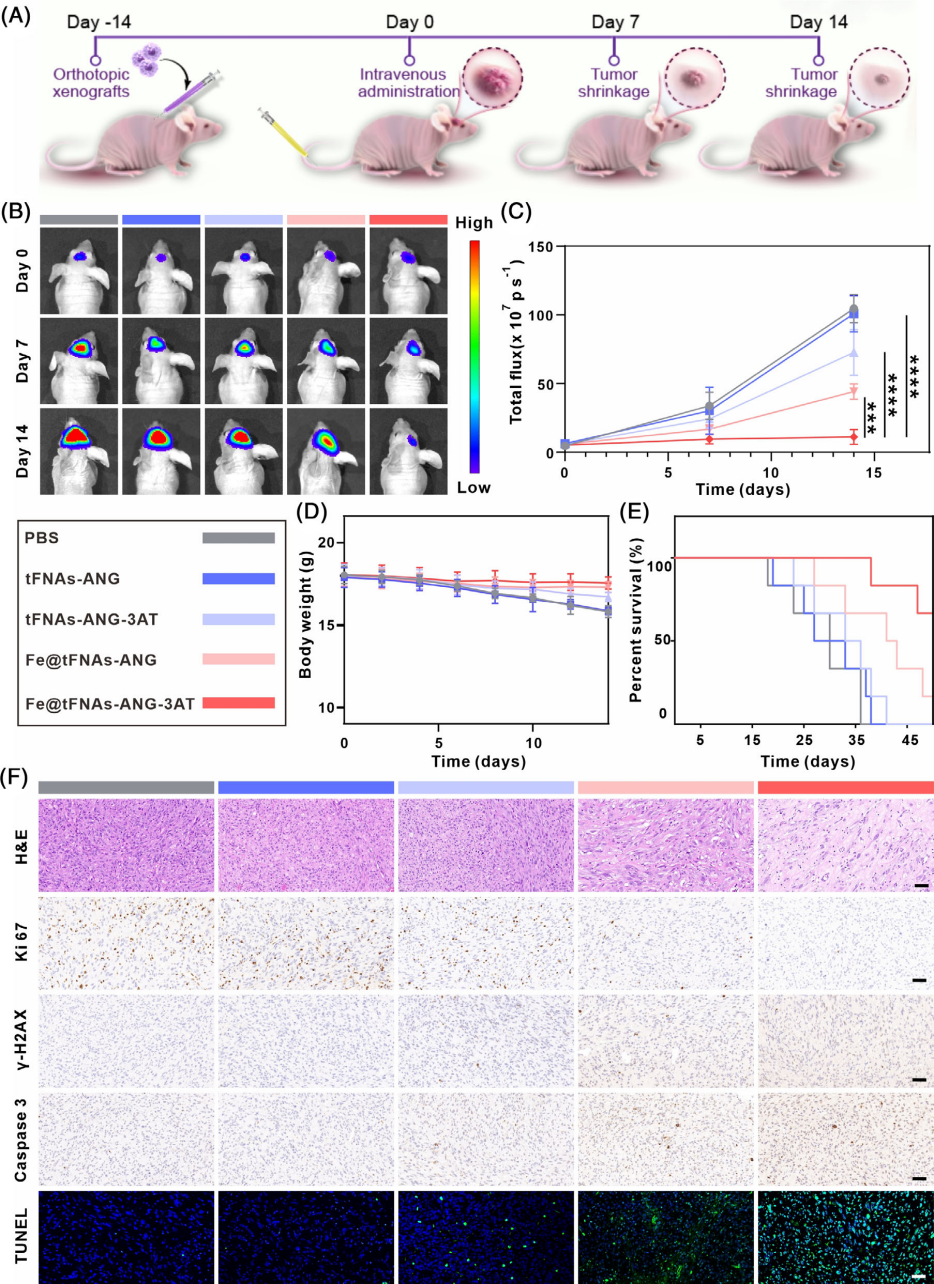
In vivo anticancer efficiency of Fe@tFNAs‐ANG‐3AT‐caused CDT against GBM. (A) Schematic depiction of the in vivo therapeutic procedure of Fe@tFNAs‐ANG‐3AT. (B) In vivo monitoring of tumour progression after intravenous injection. (*n* = 6). (C) The quantification of intensity of bioluminescent imaging signals (D, E) Weight change curves (D) and Kaplan–Meier survival curves (E) of mice after intravenous administration. Data are presented as the mean ± SD (*n* = 6). (F) Representative images of tumour sections stained with Ki67, Caspase3, H&E, γ‐H2AX, and TUNEL. Scale bars: 50 μm.

To investigate the in vivo anti‐tumour effect of Fe@tFNAs‐ANG‐3AT‐enhanced CDT on GBM, we implanted U87MG‐Luc cells into the brains of mice to establish a bioluminescent orthotopic GBM model. 14 days after cell inoculation, the tumour‐bearing mice were treated by tail vein injection of various agents (PBS, tFNAs‐ANG, tFNAs‐ANG‐3AT, Fe@tFNAs‐ANG, and Fe@tFNAs‐ANG‐3AT), respectively (Figure 5A). The tumour size was continually monitored using bioluminescent imaging. Compared with the PBS, tFNAs‐ANG, and tFNAs‐ANG‐3AT groups, tumour growth was significantly inhibited in the Fe@tFNAs‐ANG and Fe@tFNAs‐ANG‐3AT groups (Figure 5B,C). Due to the limited amount of H_2_O_2_ in the TME, the Fe@tFNAs‐ANG group only exhibited moderate anti‐tumour effects. Notably, Fe@tFNAs‐ANG‐3AT can achieve efficient CDT treatment due to its ability to increase H_2_O_2_ levels in the TME, and thus Fe@tFNAs‐ANG‐3AT‐treated mice exhibited the most pronounced tumour suppression, leading to the greatest reduction in tumour load. Throughout the treatment period, no significant weight loss was observed in mice from any treatment group (Figure 5D), confirming the satisfactory biosafety of Fe@tFNAs‐ANG‐3AT. Furthermore, the survival curve showed that the median survival time of mice administered with Fe@tFNAs‐ANG‐3AT was significantly longer compared with the other groups (Figure 5E). Subsequently, tumour tissues from each group were collected for pathological analysis (Figures 5F and S7–S10). Histological haematoxylin–eosin (HE) staining of tumour tissues treated with different formulations showed the lowest tumour cell density in the Fe@tFNAs‐ANG‐3AT treatment group. Immunohistochemical and immunofluorescence analysis revealed the most pronounced inhibition of tumour cell proliferation (Ki‐67 positive), the most severe DNA damage (γ‐H2AX positive), and a significant increase in apoptosis signals (Caspase 3 and TUNEL positive) in the Fe@tFNAs‐ANG‐3AT treatment group. These results indicate that Fe@tFNAs‐ANG‐3AT exhibits excellent anti‐tumour effects in orthotopic GBM mice and significantly prolongs their survival, making it a promising therapeutic strategy for GBM treatment.

### 2.5 In vitro and in vivo biosafety of Fe@tFNAs‐ANG‐3AT

**FIGURE 6:**
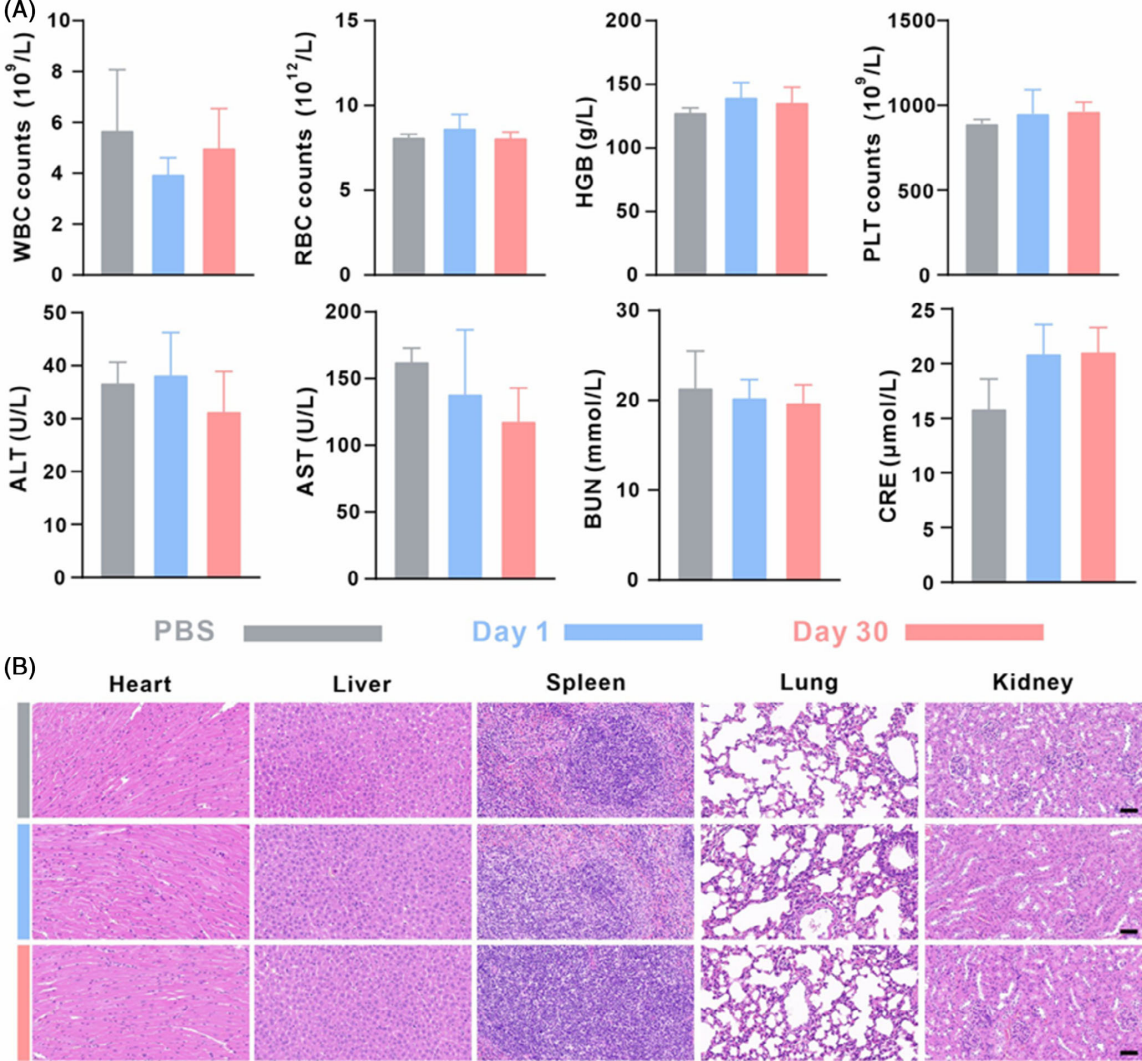
Exploration of Long‐term safety. (A) Routine blood examinations and blood biochemistry tests in healthy mice receiving PBS or Fe@tFNAs‐ANG‐3AT for 1 and 30 days. (B) Histological analysis (H&E staining) of major organs (liver, heart, lung, kidney, and spleen) from healthy mice receiving PBS or Fe@tFNAs‐ANG‐3AT for 1 and 30 days. Scale bars: 50 μm.

The safety of nanomedicine during continuous administration is the primary concern for clinical applications. To evaluate the cytotoxic effects of Fe@tFNAs‐ANG‐3AT, NHA cells (a non‐malignant cell line) were incubated with varying concentrations of Fe@tFNAs‐ANG‐3AT. Cell viability was subsequently quantified using a CCK8 assay. As illustrated in Figure S11, negligible cytotoxicity was detected, even at the concentration of 400 nM. Further, the impact of Fe@tFNAs‐ANG‐3AT on haematological and biochemical parameters was comprehensively evaluated in healthy mice through routine blood analyses and serum biochemistry assays. As shown in Figure 6A, there were no significant abnormalities observed in the blood routine parameters, including red blood cells (RBC), platelets (PLT), white blood cells (WBC), and haemoglobin (HGB). Blood biochemical indexes: the levels of liver function‐related indicators, such as aspartate aminotransferase (AST) and alanine aminotransferase (ALT), as well as kidney function‐related indicators, such as blood urea nitrogen (BUN) and creatinine (CREA), were within the normal range. In addition, H&E staining of the major organs (heart, liver, spleen, lung, and kidney) in each group of mice did not reveal significant pathological damage (Figure 6B). These results fully confirm that Fe@tFNAs‐ANG‐3AT has excellent biosafety and is a safe and effective CDT agent without toxic side effects.

## 3 CONCLUSION

To sum up, we designed a novel CDT agent (Fe@tFNAs‐ANG‐3AT), which can enhance BBB penetration, accurately target tumour cells, and increase H_2_O_2_ content in the TME to achieve efficient CDT therapy, and show satisfactory antitumor effects in orthotopic GBM mice. In addition, safety evaluation results show that Fe@tFNAs‐ANG‐3AT possesses excellent biosafety. All of these results suggest that tFNAs‐based drug delivery systems offer a promising strategy for enhancing CDT treatment in GBM.

## 4 EXPERIMENTAL SECTION

### 4.1 Synthesis of tFNAs‐ANG‐3AT and Fe@tFNAs‐ANG‐3AT

The oligo sequences were purchased from Sangon (Shanghai). tFNAs were assembled using four prefabricated ssDNAs in TM buffer (50 mM MgCl_2_, 10 mM Tris–HCl, pH 8.0) at a final concentration of 1 μM. The mixture was subjected to a thermal cycling regimen involving heating to 95°C for 10 min followed by rapid cooling to 4°C at a period of 20 min using a PCR thermal cycler (ABI, PCR system 9700). Fe@tFNAs‐ANG were prepared by adding 20 μL of 20 mM FeCl_3_·2H_2_O to 100 μL of 1 μM tFNAs‐ANG in a 200 μL PCR tube. The mixture was centrifuged at 10000 rpm, 4°C for 5 min to collect Fe@tFNAs‐ANG, which were then washed three times to remove excess Fe^3+^. tFNAs‐3AT, tFNAs‐ANG, tFNAs‐ANG‐3AT, and Fe@tFNAs‐ANG‐3AT were synthesized using the same procedure.

### 4.2 Characterizations of Fe@tFNAs‐ANG‐3AT

AFM (Multimode Nanoscope VIII, Bruker) and TEM (FEI Tecnai G2 F20 S‐TWIN electron microscope) were used to confirm the morphology and size of Fe@tFNAs‐ANG‐3AT. The composition of tFNAs‐ANG‐3AT and Fe@tFNAs‐ANG‐3AT was analysed using Energy dispersive spectroscopy (EDS, Oxford X‐MAX energy spectrometer) to determine the Fe^3+^ loading.

### 4.3 Polyacrylamide gel electrophoresis (PAGE)

PAGE (8%) analysis was performed to validate the successful assembly of the Fe@tFNAs‐ANG‐3AT. In brief, 8% PAGE was carried out in 1 × TAE buffer containing 12.5 mM MgCl_2_ at 80 V and 25°C for approximately 2.5 h. It was then stained with GelRed for 15 min and analysed using a chemiluminescence imaging system (USA, Bio‐Rad GelDoc Go).

### 4.4 Animals

Female BALB/c nude mice (4–6 weeks old) were obtained from Guangdong Medical Laboratory Animal Center and maintained in isolator cages within a pathogen‐free environment at the Experimental Animal Center of Zhujiang Hospital, Southern Medical University. The facility maintained standard environmental conditions, including 50% humidity and a temperature of 22°C. Mice were maintained on a 12‐h light/dark cycle with ad libitum access to water and standard diet. All experimental procedures were performed in accordance with protocols approved by the Laboratory Animal Ethics Committee of Zhujiang Hospital, Southern Medical University (LAEC‐2024‐092).

### 4.5 Orthotopic mouse xenografts

Anaesthetized mice were positioned in a stereotaxic device. A 6 mm cut was performed along the sagittal suture, 5 mm posterior to the junction of the inner canthus and sagittal suture, revealing the skull. With reference to a stereotactic anatomical guide, a 1 mm orifice was created 0.5 mm posterior to the bregma and 2.0 mm sideways from the midline. The stereotaxic apparatus was used to steady the mice's head. A cell suspension (5 × 10^5^ cells/mouse) was administered into the brain using a microsyringe. The needle was kept in position for 1–2 min before being slowly extracted. The incision on the scalp was sutured, and the mice were kept warm until they had completely recovered from the anaesthesia. Subsequently, they were returned to their enclosures for continuous observation.

### 4.6 BBB‐penetrating and tumour‐targeting efficiency of the Fe@tFNAs‐3AT and Fe@tFNAs‐ANG‐3AT

We established an in vitro BBB model using a permeabilized cell culture system with bEnd.3 cells to evaluate the BBB penetration ability of Fe@tFNAs‐3AT and Fe@tFNAs‐ANG‐3AT. The bEnd.3 cells were seeded on the upper chamber of a transwell and cultured to establish a monolayer with a resistance above 300 Ω·cm^2^, indicating the establishment of the in vitro BBB model.^33^, ^37^, ^38^ For the transcytosis study, The upper chamber was then supplemented with Cy5‐labelled Fe@tFNAs‐3AT and Fe@tFNAs‐ANG‐3AT. After 6 h, the culture medium from the lower chamber was collected for fluorescence intensity measurement. Additionally, we injected normal nude mice with Cy5‐labelled Fe@tFNAs‐3AT and Fe@tFNAs‐ANG‐3AT via tail vein injection. The fluorescent intensity was monitored using a small‐animal live‐subject imaging modality (IVIS) to confirm the efficient traversal of the BBB of Fe@tFNAs‐ANG‐3AT in vivo.

We assessed the tumour‐targeting ability of Fe@tFNAs‐ANG‐3AT using flow cytometry and confocal laser microscopy. U251 and U87MG cells were seeded on confocal plates and incubated at 37°C for 24 h. Following two washes with PBS, paraformaldehyde was used to fixed the cells for 15 min, stained with phalloidin and DAPI, and imaged using a laser confocal microscope (N‐SIM, Nikon, Japan). For flow cytometry analysis, U251 and U87MG cells were seeded in 6‐well plates. After the same treatment as described above, the cells were collected, washed three times with PBS, and their fluorescence intensity was quantified using a flow cytometer (Beckman Cytoflex S).

### 4.7 Mitochondrial membrane potential evaluation

U87MG cells were plated in a confocal dish including 1 mL of culture medium for 24 h. The cells were divided into five groups. The cells were treated with PBS, tFNAs‐ANG, tFNAs‐ANG‐3AT, Fe@tFNAs‐ANG, and Fe@tFNAs‐ANG‐3AT at a concentration of 400 nM for 24 h. After washing with PBS, the cells were stained with JC‐1 reagent for 20 min to evaluate the mitochondrial membrane potential. After two washes with JC‐1 buffer, cells were resuspended in fresh cell culture medium (1 mL). Finally, confocal microscopy (AX NIS‐Elements 5.4, Japan) facilitated the visualization of cell fluorescence.

### 4.8 Determination of ROS production in vitro

U87MG cells were seeded into 6‐well plates and incubated for 24 h. After that, the cells were divided into five groups, namely PBS, tFNAs‐ANG, tFNAs‐ANG‐3AT, Fe@tFNAs‐ANG, and Fe@tFNAs‐ANG‐3AT. DCFH‐DA was used as a ROS probe for detecting the intracellular ROS within U87MG cells. Then, the cells were washed with PBS to remove free samples and observed by a confocal microscope (AX NIS‐Elements 5.4, Japan).

### 4.9 Dead/live staining

U87MG cells were seeded in a 6‐well plate. After 24 h incubation, the cells were divided into five groups, the cells of each group were incubated with calcein acetoxymethyl ester (Calcein‐AM) and propidium iodide (PI) staining reagents for 10 min, and then visualized using a Nikon confocal laser microscope (AX NIS‐Elements 5.4, Japan). The live cells were stained as green fluorescence, and the dead cells as red fluorescence respectively.

### 4.10 Cell apoptosis assay

U87MG cells were seeded into 6‐well plates and treated with PBS, tFNAs‐ANG, tFNAs‐ANG‐3AT, Fe@tFNAs‐ANG, and Fe@tFNAs‐ANG‐3AT for 24 h. Then, the cells were collected, stained with 5 μL of annexin V‐FITC for 15 min followed by the addition of 10 μL of PI for 5 min at room temperature, and subsequently quantified by flow cytometer (Beckman cytoflex S).

### 4.11 In vivo antitumor efficacy

The anti‐tumour effect of Fe@tFNAs‐ANG‐3AT on U87MG‐Luc glioma‐bearing mice was investigated by tail vein injection. U87MG mice were randomly divided into five groups (*n* = 6): (1) PBS group, (2) tFNAs‐ANG group, (3) tFNAs‐ANG‐3AT group, (4) Fe@tFNAs‐ANG group, and (5) Fe@tFNAs‐ANG‐3AT group. The mice were injected intravenously with a different preparation every 3 days for a total of four injections. Subsequent live imaging sessions were conducted at seven‐day intervals. The body weight was measured every other day throughout the treatment period. After 14 days of treatment period, the mice were euthanized, and tumour samples were collected for Caspase3, H&E, Ki67, γ‐H2AX, and TUNEL staining.

### 4.12 Long‐term safety assessment

Female BALB/c nude mice were segregated into two cohorts: one administered PBS and the other administered Fe@tFNAs‐ANG‐3AT. On days 1 and 30, both groups underwent a comprehensive blood analysis encompassing platelets (PLT), haemoglobin (HGB), red blood cells (RBC), and white blood cells (WBC). In addition, blood biochemistry analyses, including blood urea nitrogen (BUN), alanine transaminase (ALT), aspartate aminotransferase (AST), and creatinine (CREA), were conducted on days 1 and 30. On days 1 and 30, haematoxylin and eosin (H&E) staining was used to examine the lungs, spleen, heart, liver, and kidneys of mice in the PBS treatment group. For the Fe@tFNAs‐ANG‐3AT treatment group, these organs were subjected to analogous scrutiny to evaluate the long‐term safety of the intervention.

### 4.13 Statistical analysis

Each experiment in the characterization of NPs was conducted a minimum of three times. Subsequently, each cellular‐level investigation was repeated at least three times. For the in vivo studies, a minimum of six mice were incorporated in each group. All results are presented as mean ± standard deviation (SD) (*n* ≥ 3). Statistical comparisons were conducted with Student's *t*‐test for two groups and one‐way ANOVA for multiple groups. *p* values indicating statistical significance were as follows: ns = not significant, *p < 0.05, **p < 0.01, ***p < 0.001, ****p < 0.0001. Data analysis was carried out using GraphPad Prism software (version 8).

## AUTHOR CONTRIBUTIONS

Xiaodie Li: Writing manuscript, Data management, Conceptualization, Technical Support, Methodology. Lei Li: Writing‐original draft preparation, Data curation, Methodology, Visualization. Xin Fu: Supervision, Resources. Shiqian Huang: Methodology, Data curation, Software. Yuhao Wang: Visualization. Yuepeng Yang: Methodology, Software. Shuqin Zhou: Methodology. Zhaowei Zou: Manuscript‐review and editing. Qing Peng: Manuscript‐reviewing & editing, Funding acquisition. Chao Zhang: Funding acquisition, Manuscript‐reviewing & editing, Project administration, Supervision.

## FUNDING INFORMATION

This study received financial support from the National Natural Science Foundation of China (Nos. 22304072 and 82373393), Science and Technology Program of Guangzhou (No. 2023A04J0060), and Guangdong Basic and Applied Basic Research Foundation (Nos. 2023A1515011042 and 2022A1515110845).

## CONFLICT OF INTEREST STATEMENT

The authors declare no competing financial interest.

## Supporting information


**Data S1.** Supporting information.

## Data Availability

The data that support the findings of this study are available on request from the corresponding author. The data are not publicly available due to privacy or ethical restrictions.
