# Supplementary material for: A novel tetrahedral framework nucleic acid‐derived chemodynamic therapy agent for effective glioblastoma treatment
Source: Cell Prolif. 2024 Aug 24;58(1):e13736. doi: 10.1111/cpr.13736 (PMC11693534; doi:10.1111/cpr.13736)
Supplement: Supplementary file 1 — Data S1. Supporting information. [file CPR-58-e13736-s001.docx]

**A Novel Tetrahedral Framework Nucleic Acid-Derived Chemodynamic Therapy Agent for effective Glioblastoma treatment**

Xiaodie Li^†^, Lei Li^†^, Xin Fu, Shiqian Huang, Yuhao Wang, Yuepeng Yang, Shuqin Zhou, Zhaowei Zou^*^, Qing Peng^*^, Chao Zhang^*^

^†^X. Li and L. Li contributed equally to this work.

X. D. Li, X. Fu, Y. H. Wang, Y. P. Yang, C. Zhang

Department of Oncology,

Zhujiang Hospital,

Southern Medical University,

Guangzhou, Guangdong, 510282, China

E-mail: [czhangsinap@163.com](mailto:czhangsinap@163.com)

L. Li, Z. W. Zou

Department of General Surgery,

Zhujiang Hospital,

Southern Medical University,

Guangzhou 510282, China

E-mail: [zouzhaowei@smu.edu.cn](mailto:zouzhaowei@smu.edu.cn)

S. Q. Huang

Clinical Research Center,

Zhujiang Hospital,

Southern Medical University,

Guangzhou 510282, China

S. Q. Zhou

Department of Anesthesiology of The Second Affiliated Hospital, School of Medicine, The Chinese University of Hong Kong, Shenzhen & Longgang District People’s Hospital of Shenzhen, Shenzhen, 518172

Q. Peng

Central Laboratory of The Second Affiliated Hospital, School of Medicine, The Chinese University of Hong Kong, Shenzhen & Longgang District People’s Hospital of Shenzhen, Shenzhen, 518172

E-mail: [pengqing@cuhk.edu.cn](mailto:pengqing@cuhk.edu.cn)


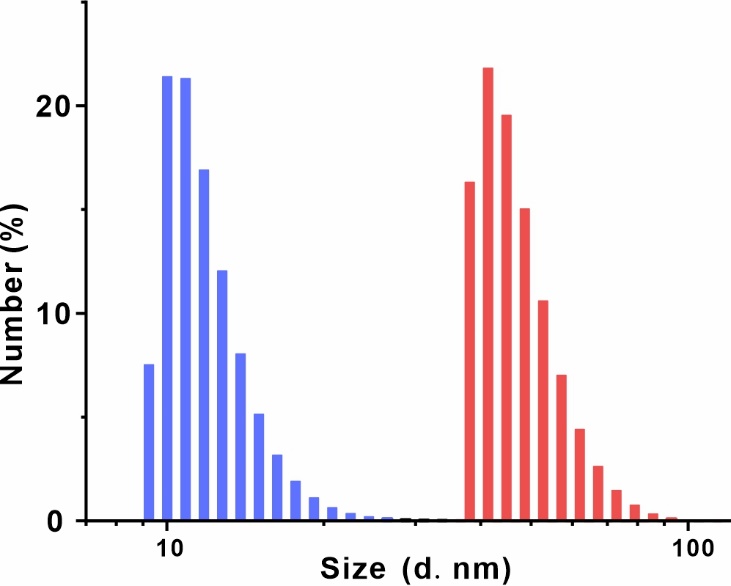


**Figure S1.** DLS of tFNAs (left) and tFNAs-ANG-3AT (right).


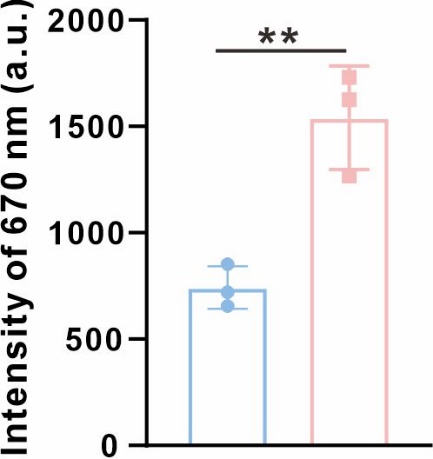


**Figure S2.** Quantitative analysis of the fluorescent intensity at 670 nm. Data are presented as the mean ± SD (n = 3, **p < 0.01).


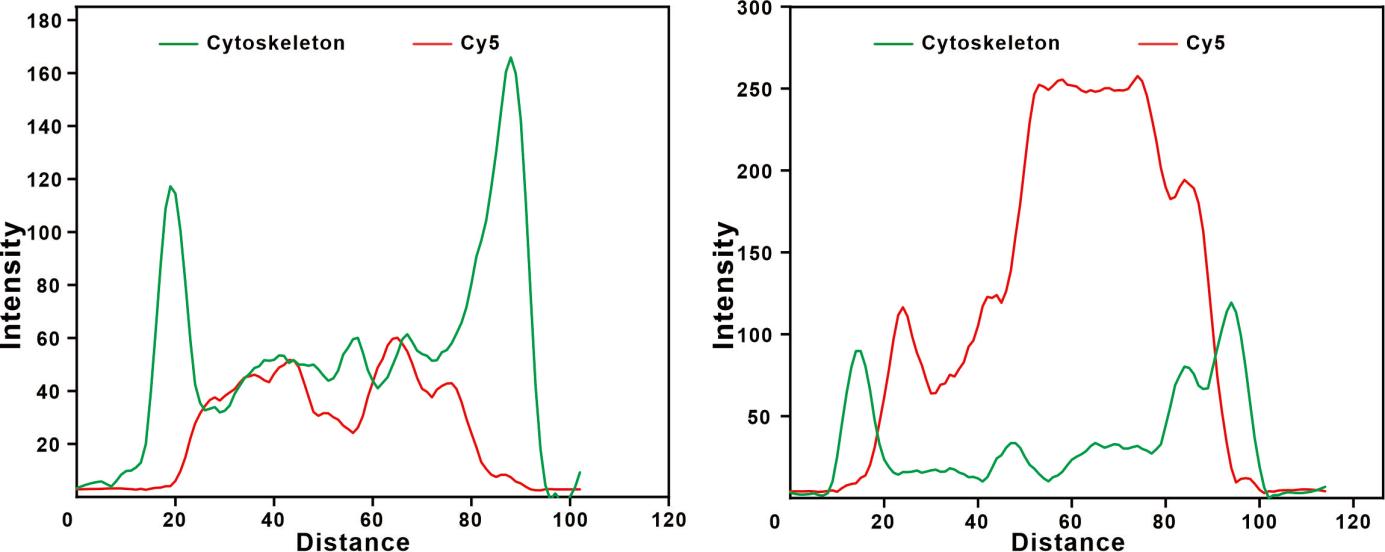


**Figure S3.** Fluorescence co-localization analysis of U87MG cells treated with Fe@tFNAs-3AT (left) and Fe@tFNAs-ANG-3AT (right) (Cy5 dosage: 400 nM, 6 h of incubation).


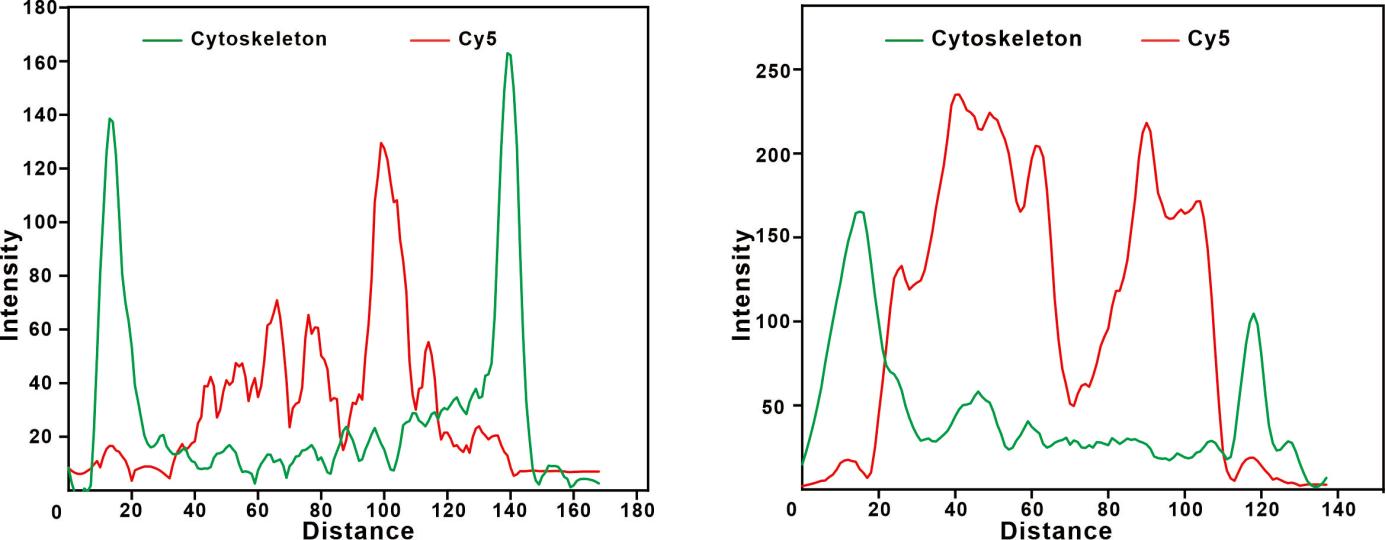


**Figure S4.** Fluorescence co-localization analysis of U251 cells treated with Fe@tFNAs-3AT (left) and Fe@tFNAs-ANG-3AT (right) (Cy5 dosage: 400 nM, 6 h of incubation).


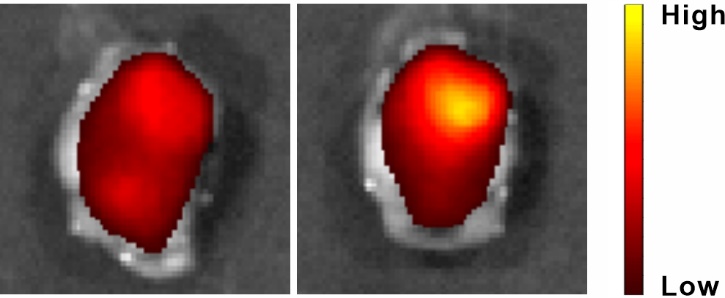


**Figure S5.** *Ex vivo* fluorescence imaging of the tumors from the mice treated with Fe@tFNAs-3AT (left) and Fe@tFNAs-ANG-3AT (right)


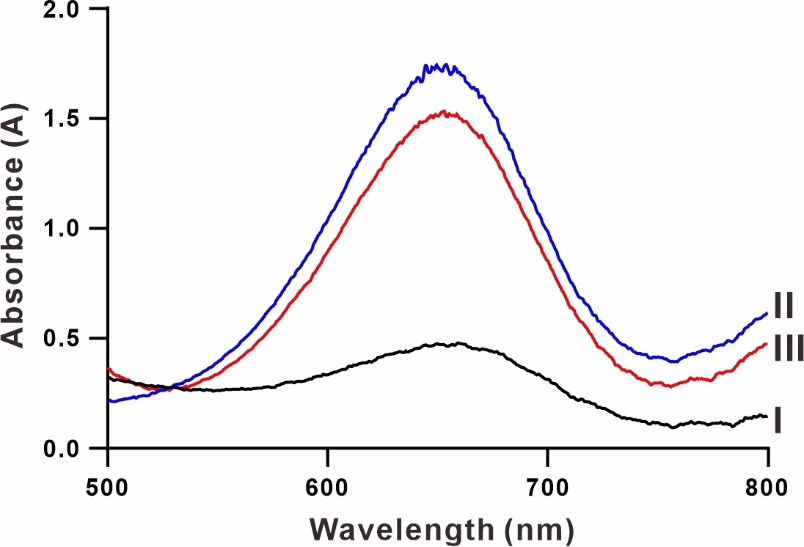


**Figure S6.** UV-vis spectra of various reaction systems after reacting with TMB (the indicator of ·OH). (I: catalase (+), 3AT (-); II: catalase (-), 3AT (-); III: catalase (+), 3AT (+)).


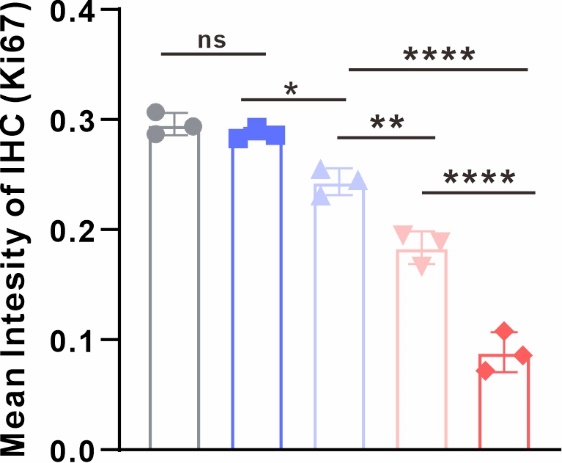


**Figure S7.** Quantitative analysis of the Ki-67 staining area fraction by Image J. ns means no significance, *p < 0.05; **p < 0.01; ****p < 0.0001.


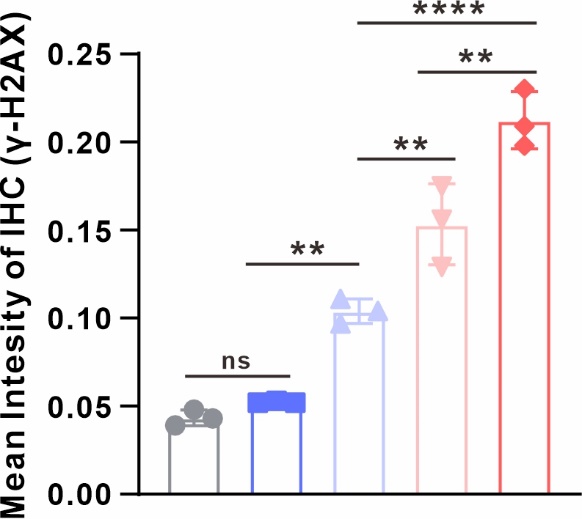


**Figure S8.** Quantitative analysis of the γ-H2AX staining area fraction by Image J. ns means no significance, *p < 0.05; **p < 0.01; ****p < 0.0001.


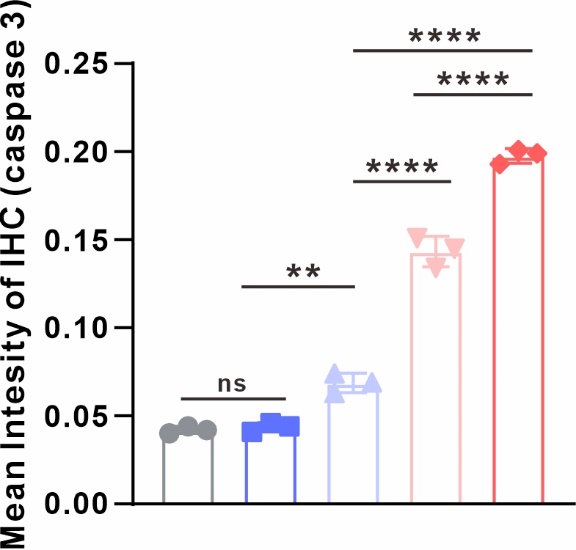


**Figure S9.** Quantitative analysis of the caspase3 staining area fraction by Image J. ns means no significance, **p < 0.01; ****p < 0.0001.


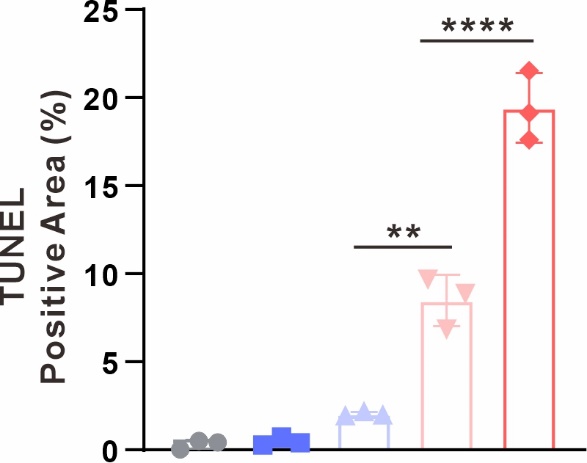


**Figure S10.** Quantitative analysis of the TUNEL staining area fraction by Image J. ns means no significance, **p < 0.01; ****p < 0.0001.


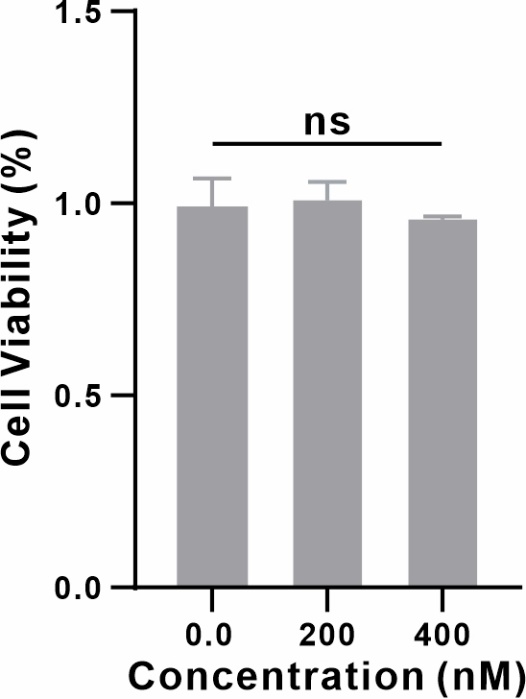


**Figure S11.** Cell viability of NHA cells after treatment with Fe@tFNAs-ANG-3AT. Data are presented as the mean ± SD (n=3, ns means no significance).
